# High-Throughput Sequencing Reveals Apple Virome Diversity and Novel Viruses in the Czech Republic

**DOI:** 10.3390/v17050650

**Published:** 2025-04-29

**Authors:** Karima Ben Mansour, Igor Koloniuk, Jana Brožová, Marcela Komínková, Jaroslava Přibylová, Tatiana Sarkisova, Jiří Sedlák, Josef Špak, Petr Komínek

**Affiliations:** 1 Ecology, Diagnostics and Genetic Resources of Agriculturally Important Viruses, Fungi and Phytoplasmas, Czech Agrifood Research Center, Drnovská 507, 161 00 Prague, Czech Republic; jana.brozova@carc.cz (J.B.); marcela.kominkova@carc.cz (M.K.); petr.kominek@carc.cz (P.K.); 2 Czech Academy of Sciences, Biology Centre, Institute of Plant Molecular Biology, Branišovská 31, 370 05 České Budějovice, Czech Republic; pribyl@umbr.cas.cz (J.P.); sarkisova@umbr.cas.cz (T.S.); spak@umbr.cas.cz (J.Š.); 3 Research and Breeding Institute of Pomology Holovousy, Ltd., Holovousy 129, 508 01 Holovousy, Czech Republic; sedlak@vsuo.cz

**Keywords:** apple, HTS, molecular variants, coinfection, ARWV1, ARWV2, recombination

## Abstract

Apple viruses pose significant threat to global apple production. In this study, HTS technology was used to investigate the apple virome in the Czech Republic. Previously reported viruses, including ACLSV, ASPV, ASGV, ApMV, AGCaV, and CCGaV, were confirmed, and near-complete genomes were assembled. Additionally, two novel viruses, ARWV1 and ARWV2 were identified for the first time in the Czech Republic. Phylogenetic analyses showed low genetic variability among ARWV2 isolates, suggesting a possible recent introduction or limited diversification. In contrast, ARWV1 isolates displayed distinct clustering in the coat protein coding region, separating symptomatic and asymptomatic samples, indicating a potential involvement of genetic determinants in symptom expression. Mixed infections were prevalent, with multiple molecular variants of ACLSV, ASPV, and AGCaV detected within individual samples, along with co-infections involving viruses from different families. Recombination analysis identified frequent recombination events in ACLSV and ASPV, often involving non-apple parental sequences, suggesting their potential for cross-host infections. Additionally, an interspecific recombination event was detected in an almond ApMV isolate, with PNRSV as a minor parent. These findings highlight the impact of agricultural practices on viral evolution and host adaptation. This study demonstrates the utility of HTS as a powerful tool for uncovering viral diversity, recombination events, and evolutionary dynamics.

## 1. Introduction

Apple (*Malus × domestica* Borkh.) is a perennial fruit crop belonging to the *Rosaceae* family. It is a hybridogenic species resulting from cultural breeding, indigenous to temperate regions [1]. Apple is one of the most economically significant fruit crops cultivated globally [2], and it is the most prevalent fruit tree species in the Czech Republic [3,4]. However, apple production is threatened by a wide range of viruses and virus-like pathogens [5] that poses a serious threat to both the quantity and quality of apple yields [6]. The apple infecting viruses belong to different families including *Betaflexiviridae*, *Tombusviridae*, *Alphaflexiviridae*, *Virgaviridae*, *Potyviridae*, *Solemoviridae*, *Bromoviridae*, and *Phenuiviridae* [7]. These viruses have the potential to induce a multitude of diseases in plants, many of which are characterised by severe symptoms [8]. Nevertheless, in certain instance, they may persist in a latent state within the host plant [9]. Some of the most economically significant viruses that have been responsible for substantial economic losses on a global scale, include apple chlorotic leaf spot (ACLSV, *Trichovirus mali*; [10]), apple stem pitting virus (ASPV, *Foveavirus mali*; [10]), and apple stem grooving virus (ASGV, *Capillovirus mali*; [10]) [11].

The propagation of apple trees is primarily achieved through vegetative techniques such as grafting, budding, cutting, and layering techniques [12], which have been identified as facilitating the spread of viruses. It is therefore crucial to diagnose these pathogens in a timely and accurate manner to prevent future economic losses. Various traditional diagnostic techniques, including serological techniques such as enzyme-linked immunosorbent assays, molecular techniques such as polymerase chain reactions (PCR and RT-PCR), and Sanger sequencing have been employed to detect apple viruses. In the Czech Republic, several viruses have been reported through these techniques, including ACLSV, ASGV, ASPV, and apple mosaic virus (ApMV, *Ilarvirus ApMV*; [10]) [13,14]. However, these techniques have limitations, as they often target only known viruses and may not detect novel viral agents and/or sequentially diverse isolates. Given the extensive diversity of apple infecting viruses is extensive and high-throughput sequencing (HTS) has emerged as a powerful tool for their unbiased detection, offering a rapid and comprehensive approach for virome characterisation [15].

The application of HTS has significantly accelerated plant virology research, enabling identification of novel viral agents. In recent years, several apple infecting viruses, including apple luteovirus 1 (ALV-1, *Luteovirus mali*; [10]), citrus concave gum associate virus (CCGaV, *Coguvirus citri*; [10]), and Solanum nigrum ilarvirus 1 (SnIV-1, *Ilarvirus SnIV1*; [10]), have been identified in private orchards, rootstock, and germplasm collections for the first time in the Czech Republic [16]. Additionally, novel viral agents, such as Malus domestica virus A (MdoVA, *Velarivirus alphamali*; [10]), and Tetranychus urticae-associated picorna-like virus 1 PK13 (TuaPV1-PK13) have been identified in apple samples through HTS [17,18].

In this study, we utilised HTS technology to characterise the virome of apple trees. The objectives were to assess the occurrence of single or multiple infections, assemble complete genomes of previously reported viruses, identify newly detected viruses, determine the phylogenetic position of Czech viral isolates within global datasets, explore recombination frequency of these viruses, and some specific molecular features of viruses found in single infections compared to publicly available sequences.

## 2. Materials and Methods

### 2.1. Sample Collection and HTS

Two sets of samples were analysed in this study (Table 1). The first set (A9, G4, H8) was derived from a collection of plants cultivated in 60-L pots in a screen house located at the virology department of Czech Agrifood Research Center at Prague-Ruzyně (CARC), where they had been maintained since 2001. These plants showed clear mosaic symptoms. The second set consisted of asymptomatic samples (S1, S4, S7, S10, S16), which were obtained from production orchards and maintained at the technical isolation facility at the Research and Breeding Institute of Pomology (Holovousy, Czech Republic) (RBIP).

For the CARC samples, total RNA was extracted from apple leaves using the Spectrum Plant Total RNA Kit (Sigma-Aldrich, St. Louis, MO, USA), following the manufacturer’s instructions. The resulting RNA was then employed for the synthesis of double-strand cDNA products, which included a ribosomal RNA depletion treatment using the QIAseq FastSelect kit (Qiagen, Hilden, Germany). The library was prepared in accordance with the Illumina DNA Prep Kit and sequenced on a NextSeq2000 instrument as paired-end reads (2 × 150 bp).

For the RBIP samples, sequencing libraries were prepared using a NEBNext Ultra II Directional RNA Library Prep kit in conjunction with NEBNext Multiplex Oligos (NEB, Ipswich, MA, USA). The library was processed on the NovaSeq 6000 platform (Admera Health Biopharma Services, New York, NY, USA) in a paired-end (2 × 150 bp) configuration.

The bioinformatic analyses were done using Geneious Prime version 2023.2 (Biomatters, Auckland, New Zealand) following with the methodology previously described [19,20]. The resulting viral genomic sequences were submitted to the GenBank database under the accession numbers (PV014885-PV014920, PV056519-PV056547).

### 2.2. Sequence and Recombination Analyses

A series of individual viral datasets was prepared in this study using both the obtained Czech isolates and those retrieved from NCBI GenBank (Table S1).

The datasets presented as complete coding regions were prepared as a concatenated ORFs and aligned using the Translator X online server (http://translatorx.co.uk/, accessed on 15 June 2024) [21].

For datasets representing specific coding regions, alignments were done using the MAFFT online server [22], followed by trimming using the BioEdit 7.2.5 software [23].

The RDP4 software was utilised to identify potential recombination events within the various datasets. The software implements seven algorithms, namely RDP, Chimaera, GENECOV, BootScan, MaxChi, SiScan, and 3Seq. For a detection to be classified as a potential recombination event, it must be supported by at least four algorithms with *p*-values < 10^−6^ [24].

### 2.3. Phylogenetic and Sequence Demarcation Analyses

The MEGA X software was used to identify the best evolutionary model, which was subsequently applied to construct the maximum-likelihood (ML) phylogenetic tree for all studied viruses. Each phylogeny was tested using 1000 bootstrap replicates [25].

The sequence demarcation tool (SDTv1.2) was used to determine pairwise nucleotide (nt) identity between different viral isolates at the nucleotides level [26].

## 3. Results

### 3.1. Virus Diversity and Co-Infections

The HTS output ranged between 27 and 44 million raw reads per a library. After quality trimming and removal of host (Malus domestica) reads, an average of 2 million reads per sample was retained for further analysis.

*De novo* assembled contigs were annotated using the BLAST module of Geneious against a custom local BLAST database (viruses and viroids), revealing a complex virome comprising eight viruses (ACLSV, ASGV, ASPV, AGCaV, ARWV1, ARWV2, CCGaV, and ApMV) from diverse families and one viroid. The identified viruses included three latent viruses from the Betaflexiviridae family namely, ACLSV, ASGV, and ASPV. In addition, viruses associated with disease symptoms were detected, including apple green crinkle associated virus (AGCaV) from the Betaflexiviridae family, three phenuivirids apple rubbery wood virus 1 (ARWV1, Rubodvirus mali; [10]), apple rubbery wood virus 2 (ARWV2, Rubodvirus prosserense; [10]), and CCGaV belonging to the Phenuiviridae family, and ApMV from the Bromoviridae family. Furthermore, one viroid, the apple hammerhead viroid (AHVd, Pelamoviroid malleusmali; [10]) from the Avsunviroidae family was also identified (Table 2).

Notably, the HTS analysis revealed several sequence variants of ACLSV, ASPV, and AGCaV, as evidenced by the closest full-length sequences used for read mapping, which showed similarity to the analysed contigs based on E-value. Two types of viral infections were found: single and multiple (Table 2). Co-infections were prevalent in both symptomatic (A9, G4, H8) and asymptomatic samples (S1, S4), with some samples harbouring up to seven different viruses. In contrast, three asymptomatic samples (S7, S10, and S16) exhibited only a single infection with ARWV1 as the only detected virus.

### 3.2. Recombination Analysis

Recombination screening of complete viral coding sequences revealed high frequency of recombination events, reaching 25%, 48% and 73% for ACLSV, ASGV and ASPV, respectively.

#### 3.2.1. ACLSV

Recombination analysis of 140 ACLSV sequences identified 24 sequences as single recombinants, while 11 sequences showed two or more recombination events (Table S2). Among 19 Czech isolates, 3 sequences (PV056525, PV056522 and PV014891) were identified as recombinants (Figure S1). The putative parental sequences of isolates PV056522 and PV056525 originated from apple hosts. A shared recombination event was detected in the Czech isolate (PV014891) and the German isolate (KX579123), with the Canadian peach isolate identified as major parental sequence. This pattern of recombination was not limited to the Czech and German isolates. Several additional NCBI-retrieved sequences also showed parental contributions from non-apple hosts, such as pear, peach, cherry, and wild apple.

#### 3.2.2. ASGV

Recombination analysis of 166 sequences (161 NCBI-retrieved sequences and 5 Czech sequences) identified 50 single recombinants and 24 isolates with multiple recombination events (Table S2). However, no recombination events were detected among the Czech ASGV isolates.

#### 3.2.3. ASPV and AGCaV

The ASPV/AGCaV dataset included 170 sequences, of which 164 were NCBI-retrieved sequences (161 ASPV sequences and 3 AGCaV) and 6 were Czech isolates (4 ASPV sequences and 2 AGCaV). The dataset was designed to investigate the relationship between both viruses by analysing potential recombination events. The RDP screening identified 58 single recombinants and 67 isolates with multiple recombination events (Table S2). Among the Czech ASPV isolates, two sequences (PV014915, PV014916) had a single recombination event. While PV014915 had both parental both parents from apple, PV014916 had a minor parental sequence from a French peach isolates (Figure S2). Similarly, several publicly available ASPV sequences were identified as recombinants (Table S2), with parental sequences originating from various hosts, including peach, pear, quince, and hawthorn. Recombination between ASPV and AGCaV was evident, with AGCaV acting as a parental contributor in 14 ASPV recombinant isolates. Among these, one Czech AGCaV sequence (PV014919) acted as a major parent in a single recombination event, while the second Czech AGCaV sequence (PV014920) was identified as a minor parent in two other events (Figure S3).

#### 3.2.4. ApMV

Recombination analysis of ApMV, a virus with a tripartite genome, showed recombination events in RNA1 and RNA2 among two NCBI-retrieved sequences. No recombination was detected in the Czech ApMV isolate (Table S2), and no events were found in RNA3.

In RNA1, the Indian isolate HE571162 from apple showed an intraspecific recombination event involving a 777 nt long fragment (Figure S4). The parental sequences were identified as apple isolates with major parent from India (OR734975) and minor parent from the USA (NC_003464).

An ApMV isolate from almond in Australia showed interspecific recombination events in both RNA1 and RNA2 (Figure S5). In RNA1, a recombinant fragment at positions 2506–2656 involved an ApMV isolate from the USA (NC_003464, major) and a PNRSV isolate from *Prunus* sp. (MZ451020, minor) from Canada. In RNA2, a larger recombinant fragment of 1317 nt was exchanged with a PNRSV isolate from *Prunus armeniaca* (KY883334, Australia), while the major parental sequence was identified as an ApMV isolate from the USA (NC_003465, apple). In addition to this, a second smaller recombinant region was detected in RNA2 (positions 1856–2045), where the major parent was the same PNRSV isolate from *Prunus armeniaca* (KY883334) and the minor parent was a Czech apple isolate (PV056546).

### 3.3. Phylogenetic and Sequence Demarcation Analyses

#### 3.3.1. Betaflexiviridae

Four viruses (ACLSV, ASGV, ASPV, AGCaV) belonging to the *Betaflexiviridae* family co-infected five samples. Multiple putative sequence variants of ACLSV, ASPV, and AGCaV were identified in each sample. Several ML phylogenetic trees were constructed to determine the position of the Czech isolates.

**Figure 1:**
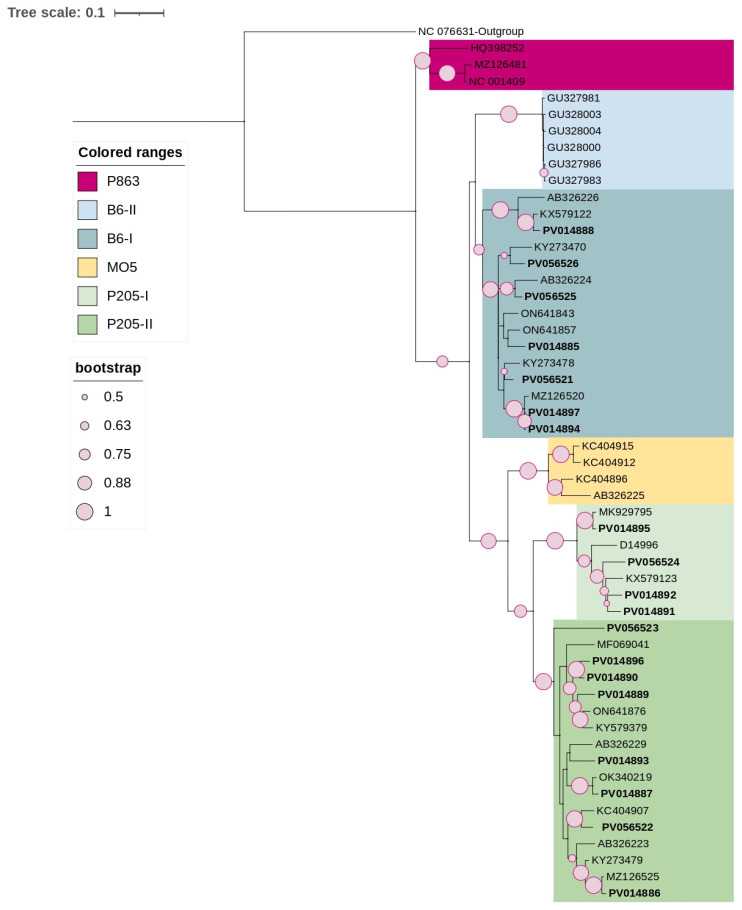
ML-phylogenetic tree constructed using the best-fitted method (K2 + G + I) and based on the complete CP coding region of 53 ACLSV isolates, including 19 Czech isolates (highlighted in bold). PCLSV (NC076631) was used as an outgroup. The tree was viewed using iTOLv7.

The ML phylogenetic tree of ACLSV (Figure 1) was constructed using 32 reference sequences representing different molecular groups (P863, B6-I, B6-II, MO5, P205-I, P205-II) as previously described by Zuļģe and her co-authors [27].

The resulting ML-phylogenetic tree (Figure 1) showed that 19 Czech isolates clustered primarily within molecular groups B6-1, P205-1, and P205-2. Sequence similarity among Czech isolates ranged from 83.2% to 99.7% at the nt level. Sample-specific sequence separation confirmed the presence of molecular variants; sequences from samples A9, H8, and S1 were divided into the B6-I clade (PV014885, PV014888, PV014897, and PV056526) and the P205-II clade (PV014886, PV014887, PV014889, PV014896, and PV056523), with high sequence variation reaching 15.4%, 13.6%, and 16.8%, respectively. Sequences from samples G4 and S4 were distributed over several clades, including clades B6-I (PV056521, PV056525, PV014894), P205-I (PV014891, PV014892, PV014895, PV056524), and P205-II (PV014890, PV014893, PV056522), with variation reaching up to 15.9%.

The ML phylogenetic tree of ASGV, constructed using the complete coding region of 87 non-recombinant isolates, revealed 15 clades (Figure S6). The Czech isolates clustered in the VIII clade with isolates from the apple hosts and diverse geographical origins, showing pairwise nt identity between 95.2% and 99.9%.

**Figure 2:**
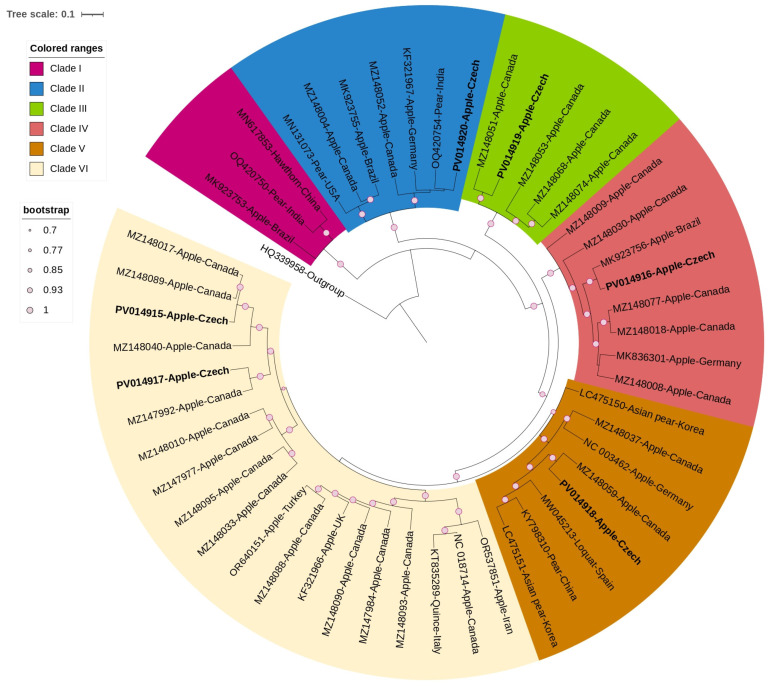
Circular maximum-likelihood phylogenetic tree using the best-fitted method (GTR + G + I) and based on 50 complete sequences of ASPV and AGCaV isolates, including 4 ASPV Czech isolates (highlighted in bold) and two AGCaV Czech isolates (highlighted in bold and marked with asteriks). Apricot latent virus (ApLV, *Foveavirus latensarmeniacae*; HQ339958) was used as an outgroup. Colours indicate different clades. The tree was viewed using iTOLv7.

The single ML phylogenetic tree was constructed using the complete concatenated coding regions of 45 non-recombinant ASPV and 5 AGCaV sequences (Figure 2), revealing six well-supported clades (bootstrap values > 0.7).

ASPV sequences were predominantly from apple samples and formed diverse clades, whereas non-apple ASPV samples consistently clustered in a separate sub-clade, suggesting host-specific grouping. Pairwise nucleotide similarities between ASPV isolates from different molecular groups ranged from 73.7 to 83.4%.

Three ASPV (PV014915-PV014917) and two AGCaV (PV014919-PV014920) isolates were found in one sample (A9), with molecular variant sequences of each virus clustering into two different clades. Among the ASPV sequences, nt similarity of the three Czech isolates ranged from 77.3% to 80.1%. One ASPV isolate (PV014916) clustered within clade IV sharing 82.3–95.3% sequence identity with other members of this clade. The other two ASPV isolates (PV014915, PV014917) clustered within clade VI sharing a similar range of sequence identity with other members of this clade. An additional ASPV (PV014918) was identified in another sample (G4).

For AGCaV, the first isolate (PV014919) clustered within clade II, sharing 80.6% to 85.9% sequence identity with other members of this clade. The second isolate (PV014920) clustered within clade III, with sequence identity ranging from 75% to 75.9% compared to other members of this clade. The two AGCaV molecular variants shared a nucleotide sequence similarity of 75.3%.

#### 3.3.2. Bromoviridae

ApMV, from the Bromoviridae family, was detected in three samples (G4, H8, S1), all of which were found in mixed infections. ML phylogenetic trees constructed using the complete coding regions revealing three clades for RNA1 (Figure S7), and RNA2 (Figure S8), while five clades were distinguished for RNA3 (Figure S9) The three Czech isolates clustered together with other NCBI-retrieved ApMV isolates, showing a low genetic variability.

#### 3.3.3. Phenuiviridae

Three viruses (ARWV1, ARWV2, and CCGaV) belonging to Phenuiviridae family were detected. ARWV1 was found in mixed infection in two samples (G4, H8) and as a single infection in three others (S7, S10, and S16). ARWV2 and CCGaV were found in mixed infection with other viruses in three (G4, H8, and S1) and two samples (S1 and S4), respectively.

**Figure 3:**
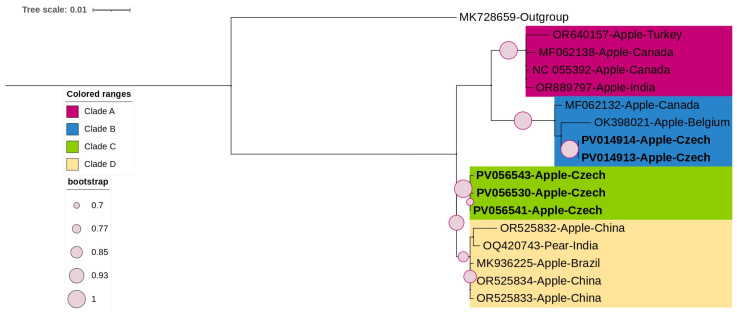
ML-phylogenetic tree constructed using the best-fitted method (T92 + I) and based on the complete coding region of coat protein of 16 ARWV1 isolates, including 5 Czech isolates (highlighted in bold). Grapevine Garan dmak virus (GGDV, *Rubodvirus armeniaense*; MK728659) was used as an outgroup. The tree was viewed using iTOLv7.

Three ARWV1 ML phylogenetic trees were constructed based on the complete coding region of replicase (Figure S10), movement (Figure S11), and coat proteins (Figure 3). The topology of the movement protein tree was largely similar to that observed for the replicase coding region, revealing four distinct clades. The five Czech ARWV1 isolates, subjects of this study, formed a distinct clade (clade C). In contrast, the ML phylogenetic tree constructed based on the coat protein coding region (Figure 3) showed a different topology, with the Czech isolates no longer clustering together. Two isolates (PV014913 and PV014914), both detected in mixed infections, clustered within clade B with isolates from Canada and Belgium. The remaining three Czech isolates (PV056530, PV056541, and PV056543), identified in single infections, remained within clade C.

A comparison of the nucleotide sequences of the CP region among the three Czech asymptomatic ARWV1 isolates (clade C) and sequences from clades A, B, and D revealed four SNPs at positions 219, 225, 385, and 696. Among these, only one SNP resulted in a non-synonymous amino acid substitution A219T unique to all three Czech isolates.

**Figure 4:**
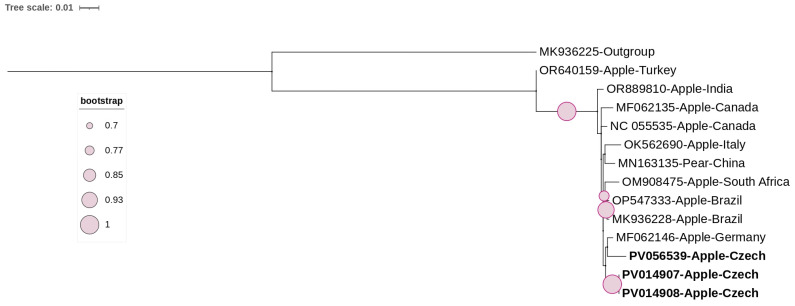
ML-phylogenetic tree constructed using the best-fitted method (T92 + I) and based on the complete coding region of coat protein of 13 ARWV2 isolates, including 3 Czech isolates (highlighted in bold). ARWV1 (MK936225) was used as an outgroup. The tree was viewed using iTOLv7.

The phylogenetic tree of ARWV2 constructed based on the complete coding region of the coat protein, included the three Czech isolates from this study along with the available NCBI complete coding regions (Figure 4). The analysis showed that all isolates clustered together, except for one Turkish isolate. The Czech isolates clustered together, showing low genetic variability, as confirmed by pairwise nucleotide identities ranging from 98% to 100%.

**Figure 5:**
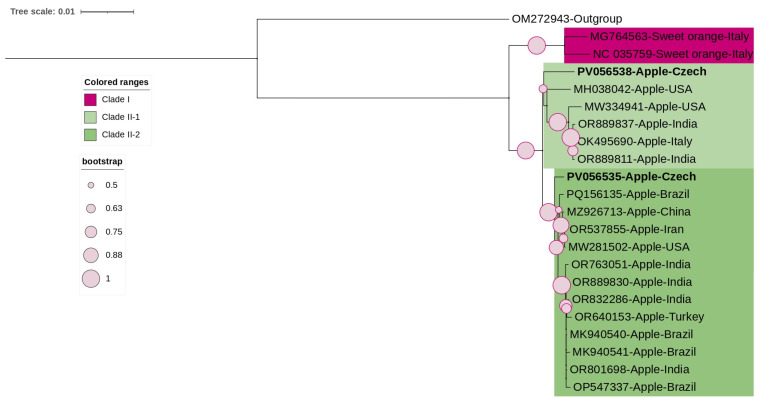
ML-phylogenetic tree constructed using the best-fitted method (T92 + I) and based on the complete coding region of RNA1 of 21 CCGaV isolates, including 2 Czech isolates (highlighted in bold). Citrus virus A (CiVA, *Coguvirus eburi*; OM272943) was used as an outgroup. The tree was viewed using iTOLv7.

The phylogenetic trees of CCGaV, constructed based on RNA1 (Figure 5), showed a clear separation between citrus and apple isolates, with citrus isolates forming a distinct clade (Clade I).

**Figure 6:**
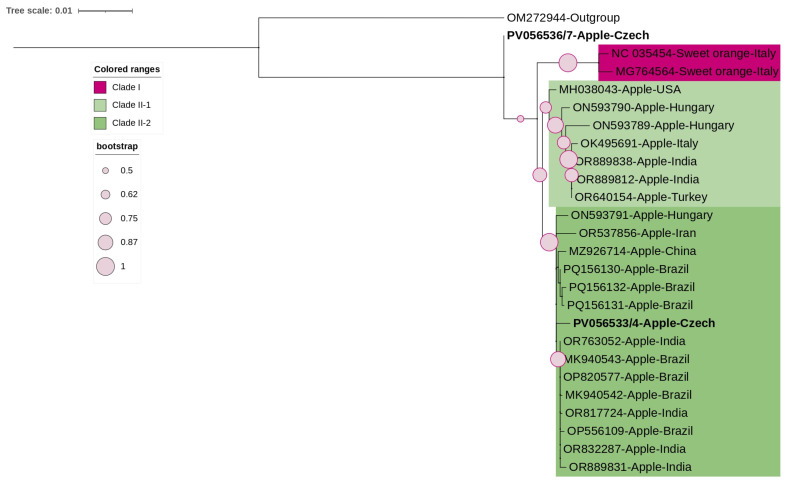
ML-phylogenetic tree constructed using the best-fitted method (T92 + I) and based on the complete coding region of MP and CP of 25 CCGaV isolates, including 2 Czech isolates (highlighted in bold). CiVA (OM272944) was used as an outgroup. The tree was viewed using iTOLv7.

Similarly, the RNA2 tree showed host-specific separation but with a different topology for the Czech isolates (Figure 6). Isolate (CCGaV-S1, PV056536) clustered separately near the outgroup, while isolate (PV05653, CCGaV-S4) grouped with other apple isolates.

## 4. Discussion

### 4.1. Application of HTS for Apple Virus Discovery

HTS has significantly advanced plant virology by enabling comprehensive detection, characterisation, and evolutionary analyses of viral genomes [28]. In this study, we used HTS to investigate apple viromes in the Czech Republic, confirming the presence of previously reported viruses, revealing new ones, and offering insights into viral diversity, recombination, and potential pathogenicity.

Our results confirmed the presence of six major apple viruses in the Czech Republic, including, ACLSV, ASPV, ASGV, ApMV, AGCaV, and CCGaV. By assembling near-complete genomes, we enabled more evolutionary analyses than previous studies relying on partial genomic sequences [13,16,29].

One example of the utility of full-genome data was observed in our segment-based phylogenetic analysis of CCGaV. The Czech isolate CCGaV-S1 showed divergent clustering between RNA1 and RNA2, a pattern that may reflect independent segment evolution, possible reassortment, or distinct selection pressures acting differently on each segment [30,31]. However, to confirm whether this pattern reflects true biological processes or sample-specific variation, additional complete genomic sequences are needed.

Beyond previously known viruses, we report for the first time the presence of ARWV1 and ARWV2 in the Czech Republic. These members of the *Phenuiviridae* family have been linked to rubbery wood disease (ARWD) in apples and are increasingly reported worldwide [32,33,34,35,36,37,38,39,40,41]. Our phylogenetic analysis showed a low nucleotide diversity among Czech ARWV2 isolates (<2%), suggesting either a recent introduction or limited local diversification, which is consistent with comparable levels (<6%), previously reported in ARWV2 isolates from pear in China [42].

### 4.2. Unclear Symptom Association of ARWV1

Our findings provide observational insights into the potential symptom associations of ARWV1 and the uncertain pathogenic role of ARWV2. In our study, ARWV1 was detected as a single infection in asymptomatic samples (S7, S10, and S16), suggesting it may persist latently or cause symptoms only under specific environment or host conditions.

By contrast, ARWV2 was previously reported as a single infection in symptomatic apple trees showing rubbery wood disease symptoms in Canada [32], suggesting a more direct role in disease expression. However, in our study, ARWV2 was never found as a single infection, and its pathogenic role remains uncertain.

The presence of ARWV1 in both asymptomatic single infections (S7, S10, and S16) and symptomatic mixed infections (G4, H8) further complicates efforts to determine its exact role in symptom development.

These observations suggest that ARWV1 may not always cause symptoms on its own, while ARWV2 is more clearly associated with disease. Further validation studies, such as grafting experiments, are needed to clarify the individual and synergistic effects of these viruses in symptom development.

### 4.3. Intra-Host and Mixed Viral Diversity

We observed extensive intra-host viral diversity, with multiple molecular variants of ACLSV, ASPV, and AGCaV detected within individual plants, ranging from two to five variants per sample. While the functional implications of such variant diversity remain uncertain, this type of infection was previously reported in other studies [43], and could result from either long-term accumulation or multiple infection events. The presence of these variants in both symptomatic and asymptomatic trees suggests that symptom expression may not correlate directly with the number or diversity of viral variants. Though biological indexing will be time and resource-intensive process, a thorough testing with different apple cultivars and viral strains is essential to clarify their relationship. Understanding this variant complexity is important, as it may not only influence symptom development but also complicate virus detection, potentially impacting disease management in perennial trees [44].

In addition to intra-host variants, we frequently observed mixed infections involving viruses from different families, supporting earlier findings that complex viromes are typical in apple trees [45,46,47]. These mixed infections may result in synergetic interactions that influence symptom severity and make it difficult to determine the role of each virus in causing symptoms.

### 4.4. Frequency of Recombination Events

One of the most important findings was the high frequency of recombination events detected in ACLSV (25%), ASGV (48%), and especially ASPV (73%), highlighting its role as a major driver of viral evolution [19,48,49]. These results align with increasing evidence that recombination contributes significantly to the adaptability and diversification of plant viruses [50,51], particularly as full-genome sequences become more available.

Among the Czech isolates, three ACLSV and two ASPV sequences were identified as recombinants, while none of the Czech ASGV isolates showed evidence of recombination. Although this differs from global pattern of recombination in ASGV, it may reflect a local bottleneck or be due to the limited sample size.

Furthermore, the involvement of non-apple (peach, pear, hawthorn) hosts as parental sources in several recombination events, suggests the potential cross-species transmission, consistent with earlier studies on apple viruses [44,47,52]. While this suggests the possibility of cross-host infections, direct evidence of inter-host viral transmission is needed to confirm it.

### 4.5. Observations on AGCaV and ASPV Relationship

The recombination and phylogenetic analyses offered additional insights into the taxonomic status of AGCaV. In our dataset, which included all publicly available AGCaV and ASPV sequences from NCBI, AGCaV was identified as a parental contributor in 14 ASPV recombinant isolates, more than previously reported [53]. Phylogenetic analysis showed that AGCaV sequences did not form a separate cluster but were intermixed with ASPV isolates [54].

This lack of distinct clustering, combined with frequent recombination events involving AGCaV, raises questions about its current classification as an unclassified foveavirus. While these findings support earlier suggestions that AGCaV may instead represent a divergent strain of ASPV [44,55], further biological evidence is needed to support any formal taxonomic revision, including serological, host range, and transmission studies.

### 4.6. Evidence of Potential Interspecific Recombination

In our analysis, we observed a rare case of possible interspecific recombination between ApMV and PNRSV, identified in an Australian almond isolate. Although this finding requires further confirmation, it raises the possibility that such recombination events may occur more frequently among ilarviruses infecting *Prunus* species.

While intraspecific recombination is well documented in ilarviruses [31,56,57], interspecific recombination has not been routinely investigated. Agricultural practices, such as the use of hybrid rootstocks in almond cultivation, could facilitate co-infection, providing opportunities for recombination between closely related viruses. Further studies are needed to assess the frequency and biological significance of such events.

## 5. Conclusions

This study used HTS to characterise the apple virome in the Czech Republic, confirming presence of known viruses and reporting ARWV1 and ARWV2 for the first time. ARWV1 was detected as a single infection in asymptomatic trees, suggesting it may persist without causing visible symptoms or only under specific conditions. In contrast, ARWV2 was found only in mixed infections, making its role in symptom development unclear.

We observed high intra-host viral diversity and frequent mixed infections, which may contribute to symptom variability. Recombination was common, particularly in ASPV, and raised questions about the classification of AGCaV as a separate species.

These findings highlight the value of full-genome analysis for understanding virus evolution, diversity, and potential disease impact in perennial crops. The frequent presence of viruses in asymptomatic trees highlights the limitations of symptom-based diagnosis and reinforces the need for virus-tested certification systems and strict phytosanitary practices in the international exchange of planting material.

## Figures and Tables

**Table 1:** Apple plants used for HTS analyses.

Apple Plant	Cultivar	Origin	Locality
A9	Rubín	CARC	Prague–Ruzyně
G4	Spartan	CARC	Prague–Ruzyně
H8	Spartan	CARC	Prague–Ruzyně
S1	Táborita	RBIP	Holovousy
S4	Golden Delicious	RBIP	Holovousy
S7	Red Boskoop	RBIP	Holovousy
S10	Golden Delicious	RBIP	Holovousy
S16	Red Boskoop	RBIP	Holovousy

**Table 2:** Identification of viruses by HTS within each sample.

Plant/Virus	ACLSV	ASGV	ASPV	AGCaV	ApMV	ARWV1	ARWV2	CCGaV	AHVd
A9	+	+	+	+					+
G4	+	+	+	+	+	+	+		+
H8	+	+	+	+	+	+	+		+
S1	+	+	+	+	+		+	+	
S4	+	+	+	+				+	
S7						+			
S10						+			
S16						+			

(The symbol (+) indicates that the virus is present).

## Data Availability

The virus genomic sequence obtained in the present work was deposited in the GenBank database of the National Center for Biotechnology Information (NCBI) under accession numbers (PV014885- PV014920). Further data that support the findings of this study are available from the corresponding author upon request.

## Supplementary Materials

The following supporting information can be downloaded at: https://www.mdpi.com/article/10.3390/v17050650/s1, Figure S1. Recombination events in three Czech ACLSV sequences (PV056525, PV056522, and PV014891) detected using RDP4. Each panel shows a recombinant sequence with its putative major and minor parents. The top bars illustrate genome-wide parental contributions, with regions derived from the major parent in dark blue and from the minor parent in green. Recombination events are shaded in pink. The bottom plots present graphical BootScan analysis, which visualise similarities between the recombinant and its potential parents, with relationships colour-coded as shown in the figure; Figure S2. Recombination events in two Czech ASPV sequences (PV014915 and PV014916) detected using RDP4. Each panel shows a recombinant sequence with its putative major and minor parents. The top bars illustrate genome-wide parental contributions, with regions derived from the major parent in dark blue and from the minor parent in green. Recombination events are shaded in pink. The bottom plots present graphical BootScan analysis, which visualise similarities between the recombinant and its potential parents, with relationships colour-coded as shown in the figure; Figure S3. Recombination events in two ASPV sequences (KF915809 and MZ147985) detected using RDP4, with the Czech AGCaV isolate acting as a parent contributor. Each panel shows a recombinant sequence with its putative major and minor parents. The top bars illustrate genome-wide parental contributions, with regions derived from the major parent in dark blue and from the minor parent in green. Recombination events are shaded in pink. The bottom plots present graphical BootScan analysis, which visualise similarities between the recombinant and its potential parents, with relationships colour-coded as shown in the figure; Figure S4. Intraspecific recombination event in one NCBI-retrieved ApMV sequence (HE571162) detected using RDP4. Each panel shows a recombinant sequence with its putative major and minor parents. The top bars illustrate genome-wide parental contributions, with regions derived from the major parent in dark blue and from the minor parent in green. Recombination events are shaded in pink. The bottom plots present graphical BootScan analysis, which visualise similarities between the recombinant and its potential parents, with relationships colour-coded as shown in the figure; Figure S5. Recombination events in one NCBI-retrieved ApMV sequence detected using RDP4. (a) shows intraspecific recombination in RNA1 (KY883318). (b) shows multiple interspecific recombination events in RNA2 (KY883319). Each panel shows a recombinant sequence with its putative major and minor parents. The top bars illustrate genome-wide parental contributions, with regions derived from the major parent in dark blue and from the minor parent in green. Recombination events are shaded in pink. The bottom plots present graphical BootScan analysis, which visualise similarities between the recombinant and its potential parents, with relationships colour-coded as shown in the figure; Figure S6. Circular maximum-likelihood phylogenetic tree constructed using the best-fitted method (GTR + G + I) and based on 86 complete sequences of ASGV, including 5 ASGV Czech isolates (highlighted in bold). Breadfruit capillovirus 1 (BCV1, MW328738) was used as an outgroup. Colours indicate different clades. Tree was viewed using iTOL; Figure S7. ML-phylogenetic tree constructed using the best-fitted method (TN93 + G) and based on the complete coding region of RNA1 of 18 ApMV isolates, including 3 Czech isolates (highlighted in bold). PNRSV (MZ451020) was used as an outgroup. The tree was viewed using iTOL; Figure S8. ML-phylogenetic tree constructed using the best-fitted method (TN93 + G) and based on the complete coding region of RNA2 of 13 ApMV isolates, including 3 Czech isolates (highlighted in bold). PNRSV (KY883334) was used as an outgroup. The tree was viewed using iTOL; Figure S9. ML-phylogenetic tree constructed using the best-fitted method (TN93 + G) and based on the complete coding region of RNA3 of 23 ApMV isolates, including 3 Czech isolates (highlighted in bold). PNRSV (MH730940) was used as an outgroup. The tree was viewed using iTOL; Figure S10. ML-phylogenetic tree constructed using the best-fitted method (GTR + I) and based on the complete coding region of replicase of 15 ARWV1 isolates, including 5 Czech isolates (highlighted in bold). ARWV2 (MF062128) was used as an outgroup. The tree was viewed using iTOL; Figure S11. ML-phylogenetic tree constructed using the best-fitted method (T92 + I) and based on the complete coding region of movement protein of 17 ARWV1 isolates, including 5 Czech isolates (highlighted in bold). ARWV2 (OR640158) was used as an outgroup. The tree was viewed using iTOL; Table S1: List of sequences used for recombination analyses; Table S2: Recombination analysis.
