# Supplementary material for: High-Throughput Sequencing Reveals Apple Virome Diversity and Novel Viruses in the Czech Republic
Source: Viruses. 2025 Apr 29;17(5):650. doi: 10.3390/v17050650 (PMC12115486; doi:10.3390/v17050650)
Supplement: Supplementary file 1 [file viruses-17-00650-s001.zip › Supplementary figures S1-S11.pdf]

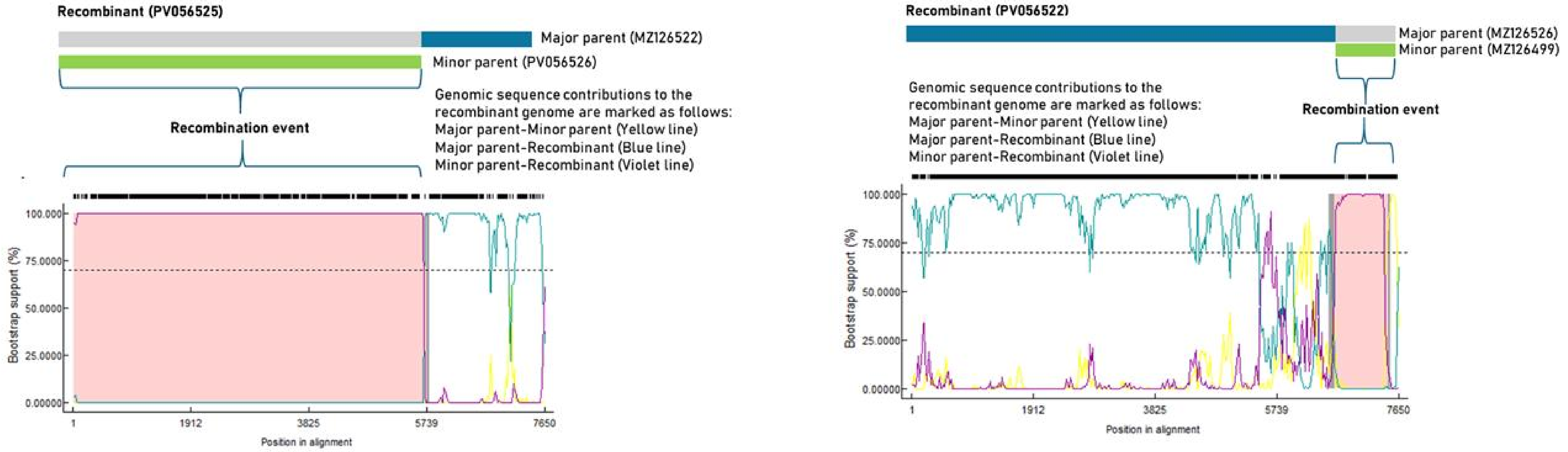

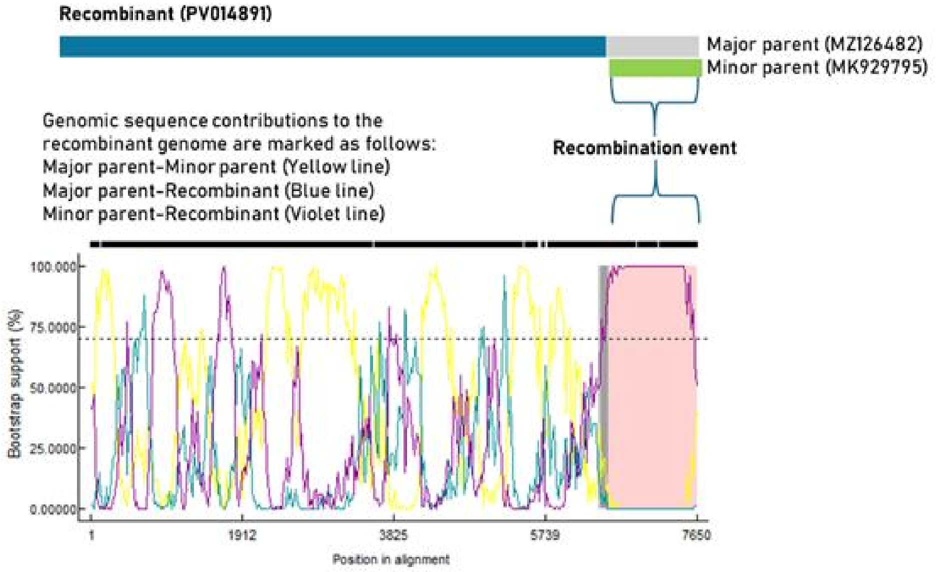

Figure S1. Recombination events in three Czech ACLSV sequences (PV056525, PV056522, and PV014891) detected using RDP4. Each panel shows a recombinant sequence with its putative major and minor parents. The top bars illustrate genome-wide parental contributions, with regions derived from the major parent in dark blue and from the minor parent in green. Recombination events are shaded in pink. The bottom plots present graphical BootScan analysis, which visualise similarities between the recombinant and its potential parents, with relationships colour-coded as shown in the figure.

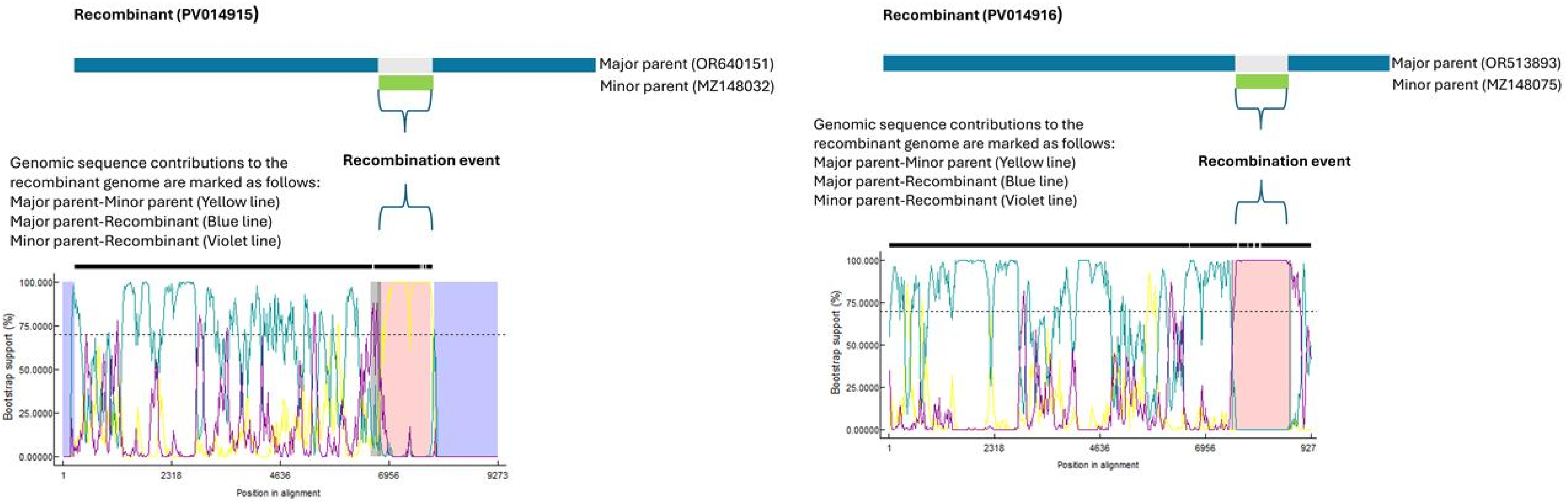

Figure S2. Recombination events in two Czech ASPV sequences (PV014915 and PV014916) detected using RDP4. Each panel shows a recombinant sequence with its putative major and minor parents. The top bars illustrate genome-wide parental contributions, with regions derived from the major parent in dark blue and from the minor parent in green. Recombination events are shaded in pink. The bottom plots present graphical BootScan analysis, which visualise similarities between the recombinant and its potential parents, with relationships colour-coded as shown in the figure.

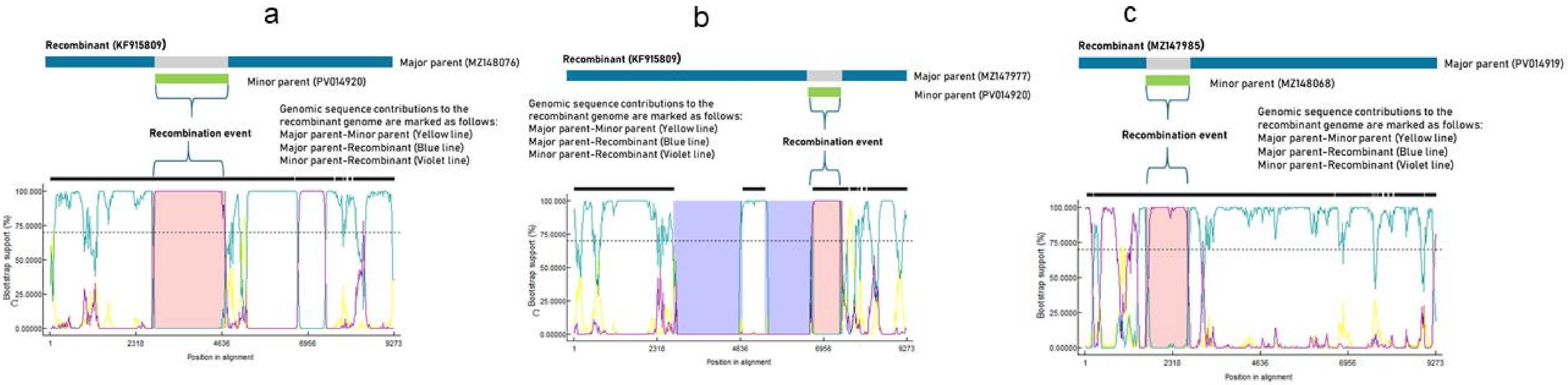

Figure S3. Recombination events in two ASPV sequences (KF915809 and MZ147985) detected using RDP4, with the Czech AGCaV isolate acting as a parent contributor. Each panel shows a recombinant sequence with its putative major and minor parents. The top bars illustrate genome-wide parental contributions, with regions derived from the major parent in dark blue and from the minor parent in green. Recombination events are shaded in pink. The bottom plots present graphical BootScan analysis, which visualise similarities between the recombinant and its potential parents, with relationships colour-coded as shown in the figure.

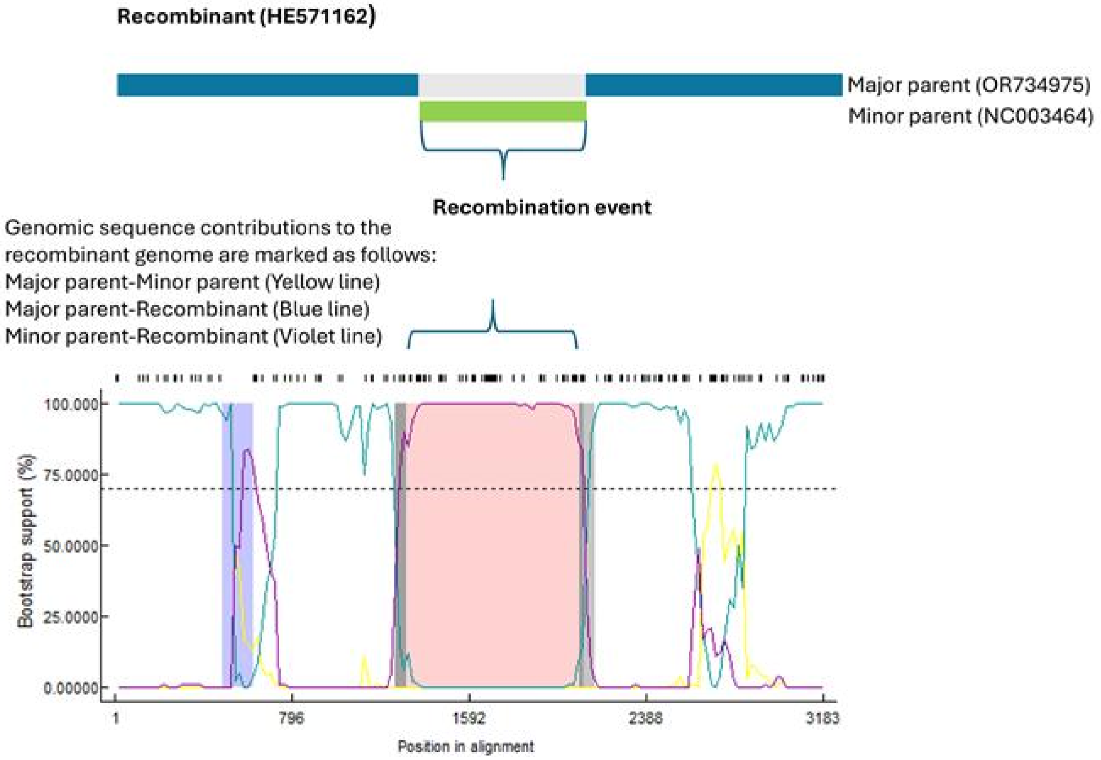

Figure S4. Intraspecific recombination event in one NCBI-retrieved ApMV sequence (HE571162) detected using RDP4. Each panel shows a recombinant sequence with its putative major and minor parents. The top bars illustrate genome-wide parental contributions, with regions derived from the major parent in dark blue and from the minor parent in green. Recombination events are shaded in pink. The bottom plots present graphical BootScan analysis, which visualise similarities between the recombinant and its potential parents, with relationships colour-coded as shown in the figure.

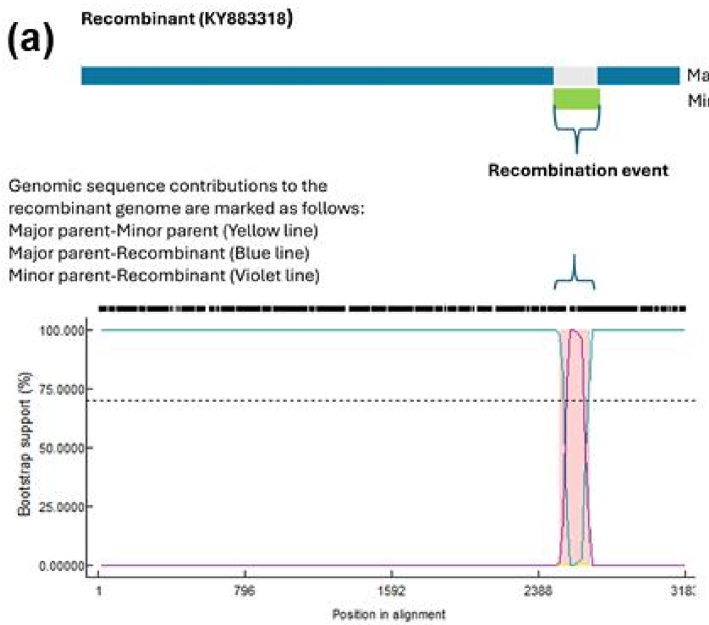

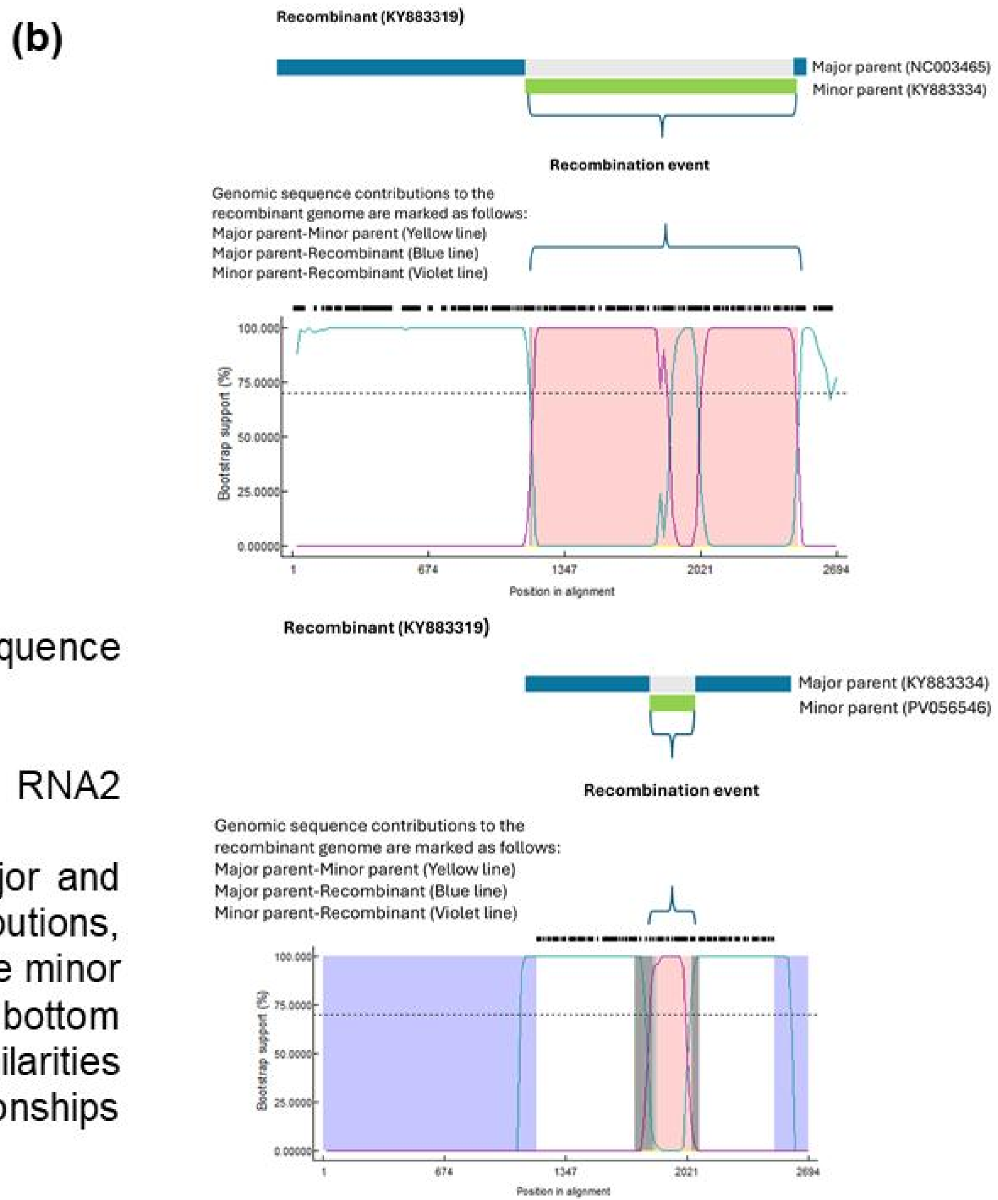

Figure S5. Recombination events in one NCBI-retrieved ApMV sequence detected using RDP4.
(a) shows intraspecific recombination in RNA1 (KY883318).
(b) shows multiple interspecific recombination events in RNA2 (KY883319).
Each panel shows a recombinant sequence with its putative major and minor parents. The top bars illustrate genome-wide parental contributions, with regions derived from the major parent in dark blue and from the minor parent in green. Recombination events are shaded in pink. The bottom plots present graphical BootScan analysis, which visualise similarities between the recombinant and its potential parents, with relationships colour-coded as shown in the figure.

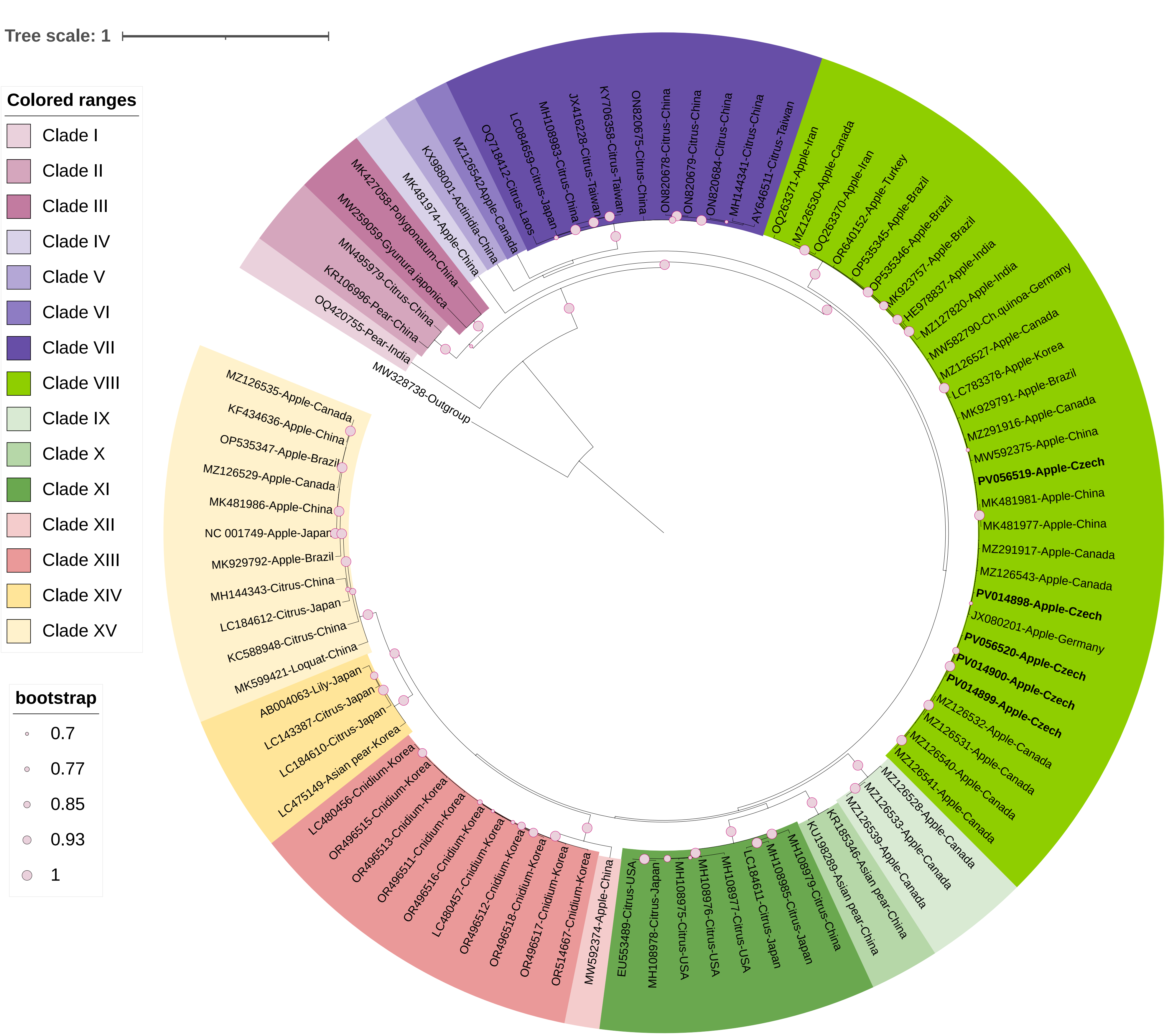

Figure S6. Circular maximum-likelihood phylogenetic tree constructed using the best-fitted method (GTR+G+I) and based on 86 complete sequences of ASGV, including 5 ASGV Czech isolates (highlighted in bold). Breadfruit capillovirus 1 (BCV1, MW328738) was used as an outgroup. Colours indicate different clades. The tree was viewed using iTOL.

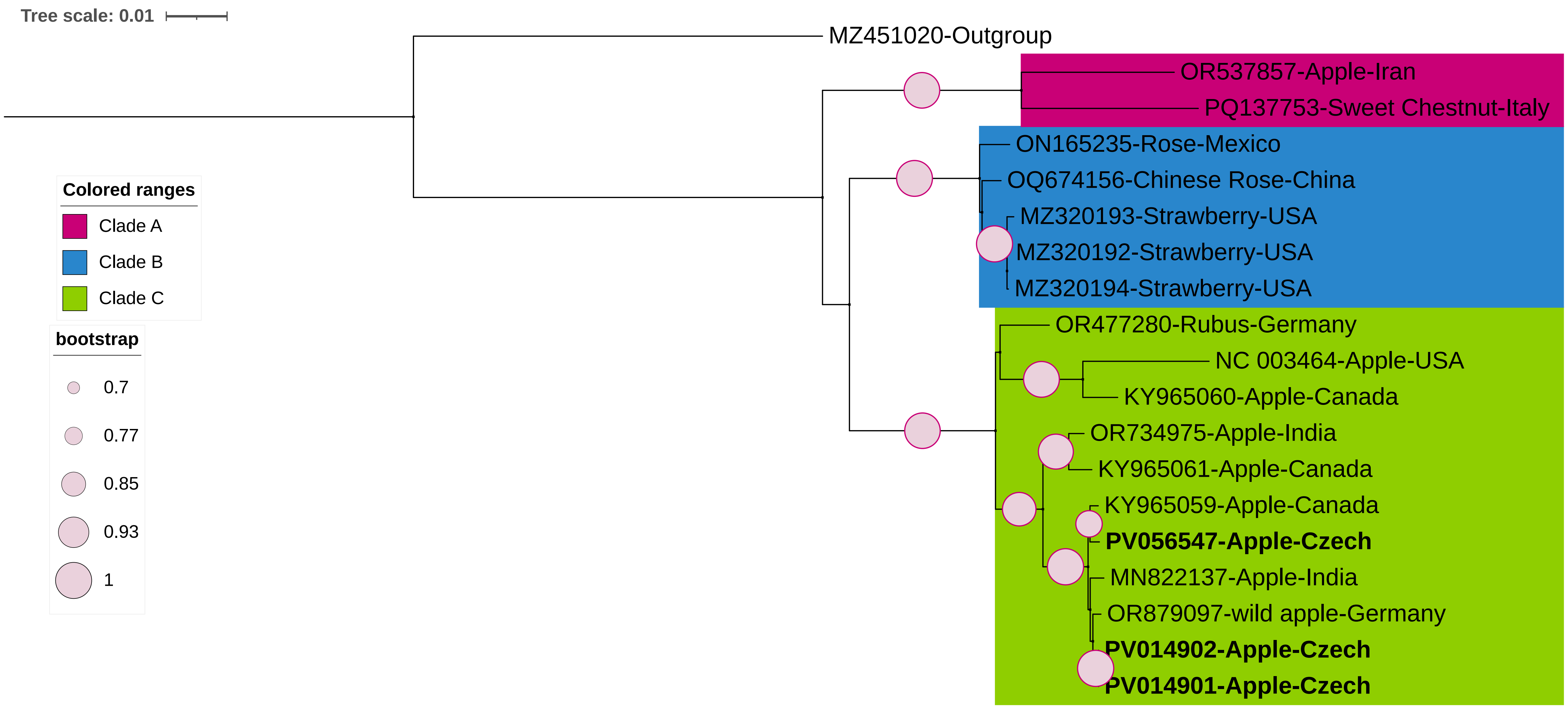

Figure S7. ML-phylogenetic tree constructed using the best-fitted method (TN93+G) and based on the complete coding region of RNA1 of 18 ApMV isolates, including 3 Czech isolates (highlighted in bold). PNRSV (MZ451020) was used as an outgroup. The tree was viewed using iTOL.

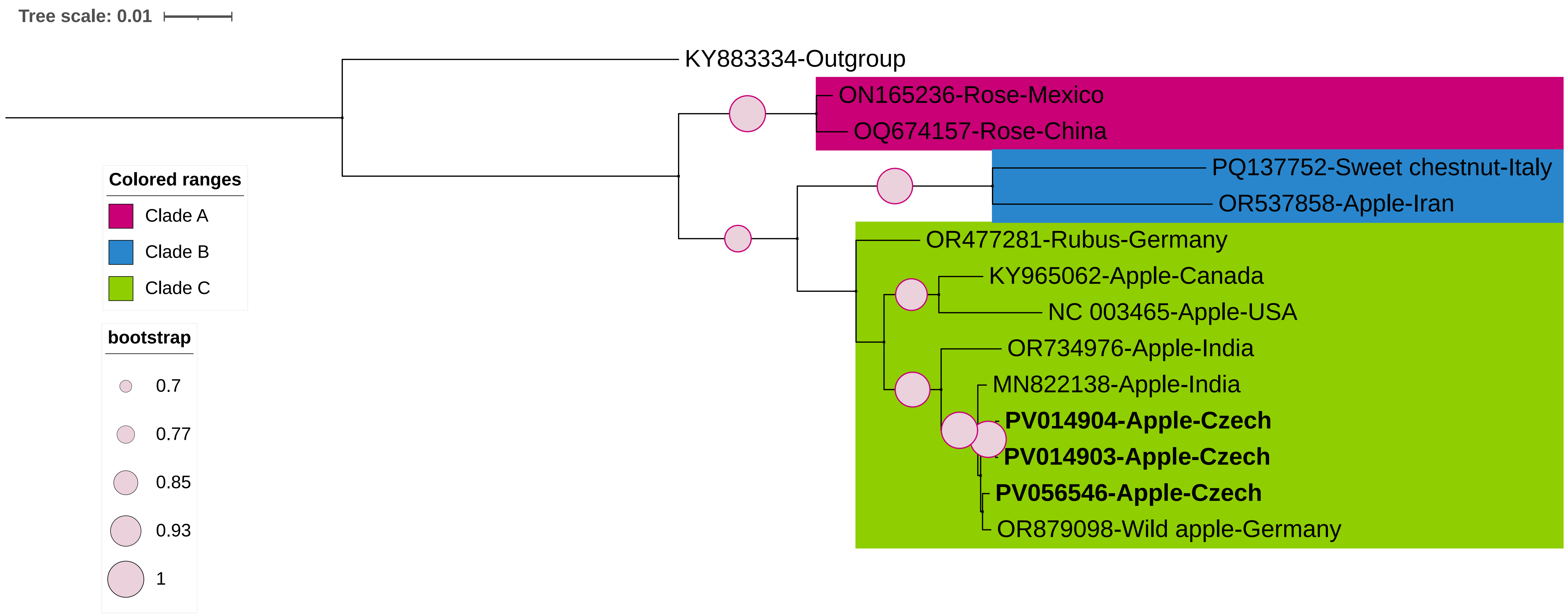

Figure S8. ML-phylogenetic tree constructed using the best-fitted method (TN93+G) and based on the complete coding region of RNA2 of 13 ApMV isolates, including 3 Czech isolates (highlighted in bold). PNRSV (KY883334) was used as an outgroup. The tree was viewed using iTOL.

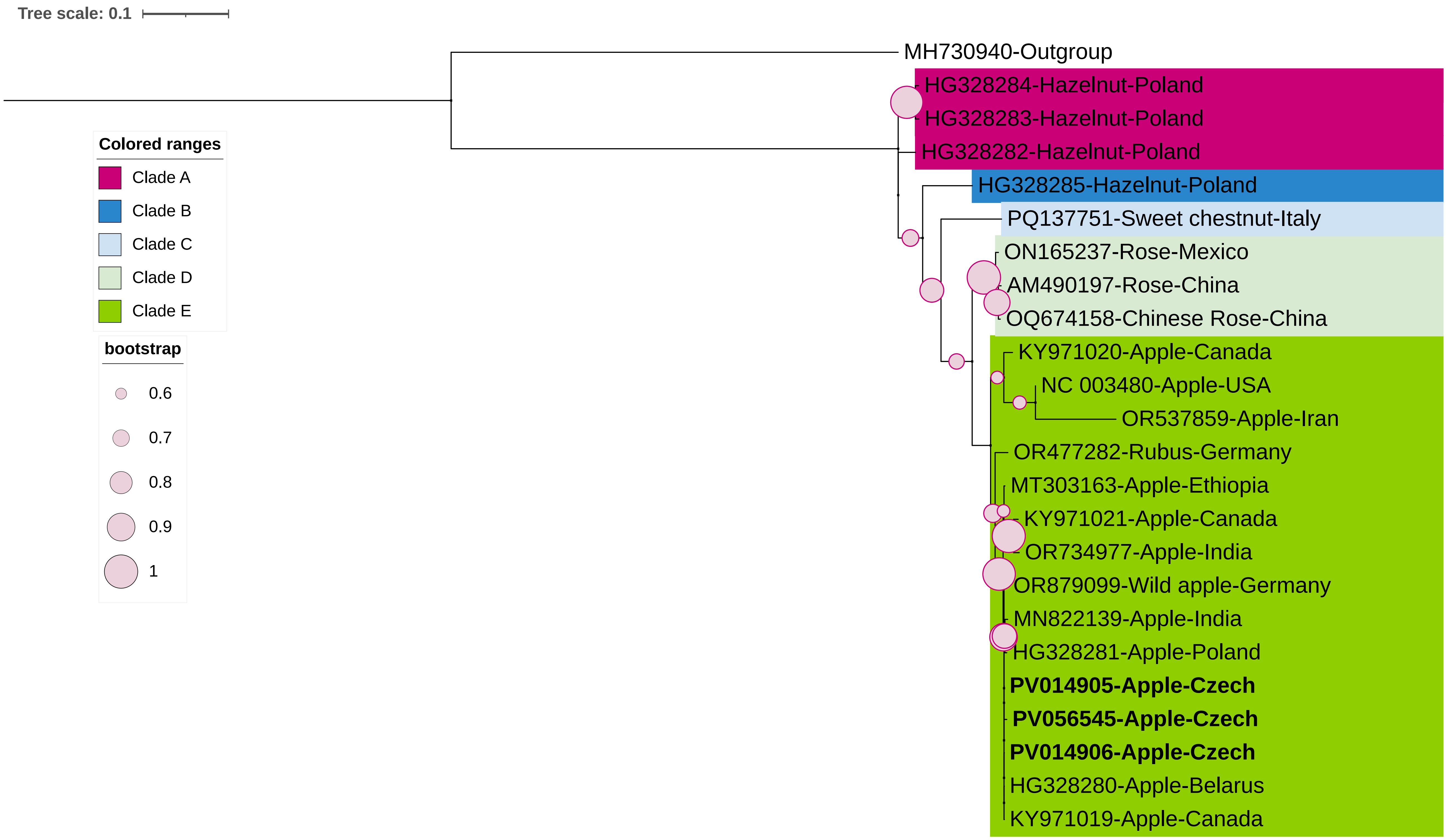

Figure S9. ML-phylogenetic tree constructed using the best-fitted method (TN93+G) and based on the complete coding region of RNA3 of 23 ApMV isolates, including 3 Czech isolates (highlighted in bold). PNRSV (MH730940) was used as an outgroup. The tree was viewed using iTOL.

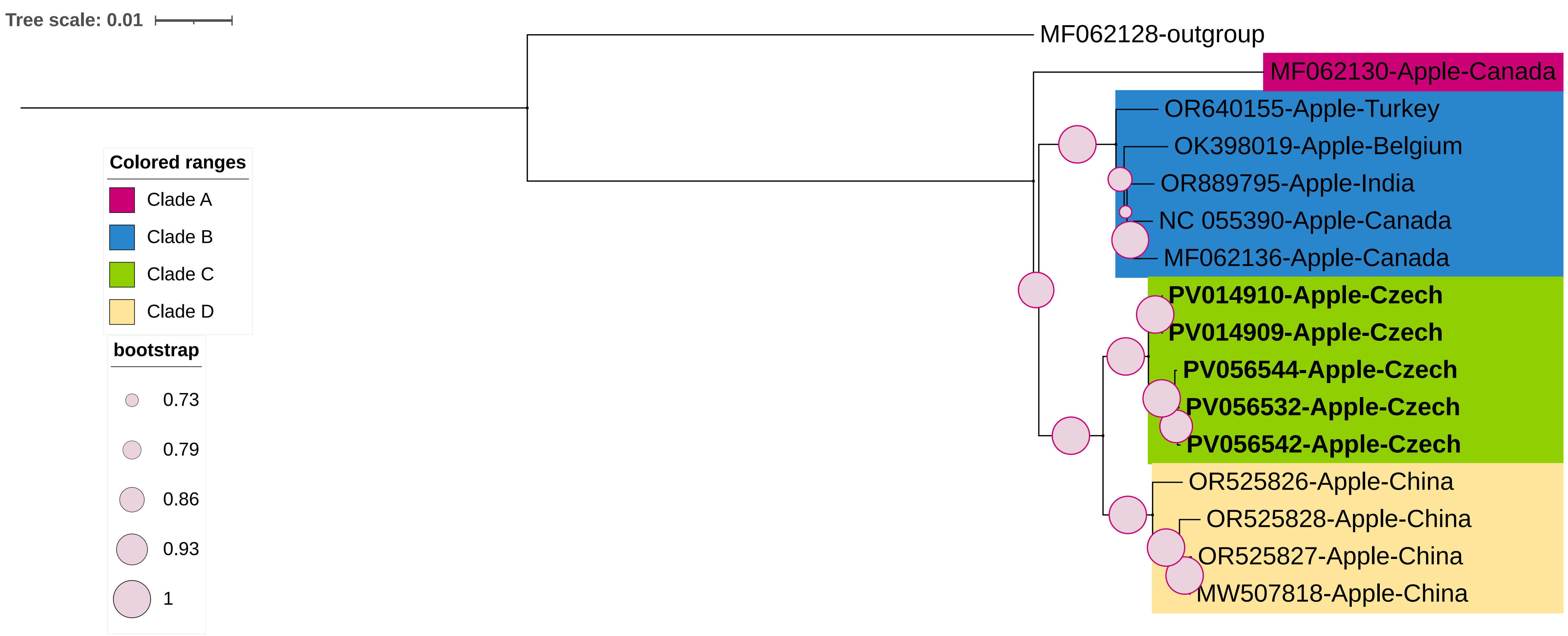

Figure S10. ML-phylogenetic tree constructed using the best-fitted method (GTR+I) and based on the complete coding region of replicase of 15 ARWV1 isolates, including 5 Czech isolates (highlighted in bold). ARWV2 (MF062128) was used as an outgroup. The tree was viewed using iTOL.

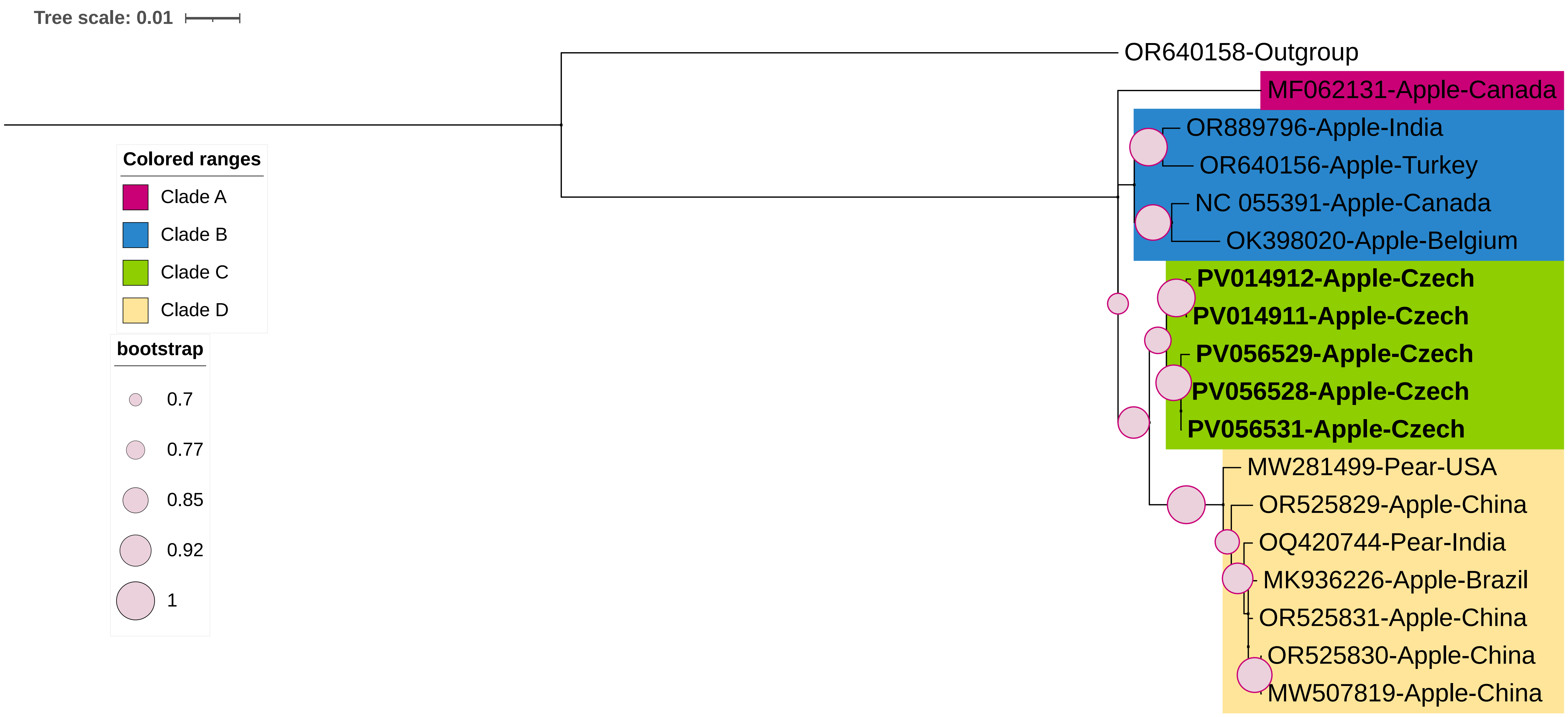

Figure S11. ML-phylogenetic tree constructed using the best-fitted method (T92+I) and based on the complete coding region of movement protein of 17 ARWV1 isolates, including 5 Czech isolates (highlighted in bold). ARWV2 (OR640158) was used as an outgroup. The tree was viewed using iTOL
